# Seaweed’s Bioactive Candidate Compounds to Food Industry and Global Food Security

**DOI:** 10.3390/life10080140

**Published:** 2020-08-06

**Authors:** Adriana Leandro, Diana Pacheco, João Cotas, João C. Marques, Leonel Pereira, Ana M. M. Gonçalves

**Affiliations:** 1 Department of Life Sciences, Marine and Environmental Sciences Centre (MARE), University of Coimbra, 3000-456 Coimbra, Portugal; adrianaleandro94@hotmail.com (A.L.); dianampacheco96@gmail.com (D.P.); jcotas@gmail.com (J.C.); jcmimar@ci.uc.pt (J.C.M.); leonel@bot.uc.pt (L.P.); 2 Department of Biology and CESAM, University of Aveiro, 3810-193 Aveiro, Portugal

**Keywords:** seaweed, nutritional, human food, sub-products, food industry, benefits, concerns, safety, food quality, regulation

## Abstract

The world population is continuously growing, so it is important to keep producing food in a sustainable way, especially in a way that is nutritious and in a sufficient quantity to overcome global needs. Seaweed grows, and can be cultivated, in seawater and generally does not compete for arable land and freshwater. Thus, the coastal areas of the planet are the most suitable for seaweed production, which can be an alternative to traditional agriculture and can thus contribute to a reduced carbon footprint. There are evolving studies that characterize seaweed’s nutritional value and policies that recognize them as food, and identify the potential benefits and negative factors that may be produced or accumulated by seaweed, which are, or can be, dangerous for human health. Seaweeds have a high nutritional value along with a low caloric input and with the presence of fibers, proteins, omega 3 and 6 unsaturated fatty acids, vitamins, and minerals. Moreover, several seaweed sub-products have interesting features to the food industry. Therefore, the focus of this review is in the performance of seaweed as a potential alternative and as a safe food source. Here described is the nutritional value and concerns relating to seaweed consumption, and also how seaweed-derived compounds are already commercially explored and available in the food industry and the usage restrictions to safeguard them as safe food additives for human consumption.

## 1. Introduction

The world population is growing to a level where the current system of food production is not capable of regularly providing food for over 9 billion people, and it is expected to grow even more, while trying to mitigate climate change and environmental pollution. Food demand is estimated to grow to at least 70% of the current food production. Intensive agriculture has led to an over exploitation of arable land and reduced access to freshwater, consequently exacerbating climatic change and having a high impact on the environment, which may be the trigger to shift research towards developing new and sustainable food sources or advancing towards under-exploited crops. There has been research developed aimed at achieving new ecological and efficient food vegetable culture methods, such as hydroponics or seaweed farming [1,2]. Unlike hydroponic cultures, which cause a negative environmental impact, by pressing freshwater reserves, seaweed cultivation only requires the seawater that is available in the open ocean, making it more feasible.

Having in mind climate change events and the increase in negative impacts on the environment, the United Nations 2030 Agenda launched 17 Sustainable Development Goals (SDGs) that changed the tenacity level to pressure United Nations member states to have a more incisive approach in the “blueprint for peace and prosperity for people and the planet”. Food production, climate change, oceans, and aquatic ecosystems will play a key role in the SDG’s achievements. In particular, SDG 2 aims to end all forms of hunger, achieve food security, improve nutrition, and promote sustainable agriculture; SDG 3 ensures healthy lives and promotes well-being for all people at all ages; SDG 12 ensures sustainable consumption and production patterns; SDG 13 highlights the urgent action required to mitigate climate change and its impacts; and SDG 14 aims to conserve and to sustainably manage the use of the oceans, seas, and marine resources for sustainable development. These SDGs promote a more eco-sustainable food production, with a reduction in negative effects and a regular food chain based on food security [3,4].

One of the most under-exploited crops are seaweeds, commonly considered as sea vegetables. However, they are increasingly being explored and are gaining more interest due to their cultivation offshore, which prevents the utilization of land, and so can be cultivated from distinct locations and need not compete for spots of agricultural land. Given this, seaweeds have high potential to be an important alternative to the world’s vegetable diet, thus augmenting the food supply chain. In this offshore cultivation technique, there is no need to use additional nutrients, unlike agriculture that is highly based on irrigated crops, which come at a great cost to the environment and collectively leave an enormous carbon footprint and the eutrophication of aquatic systems [5,6,7,8,9]. Moreover, at this point, seaweeds can edge these problems, because they are marine carbon fixators and have the potential to help in the bioremediation of eutrophic waters [10,11,12,13].

The majority of seaweeds are edible and are a high and sustainable source of macro- and micronutrients in the human diet. Contrary to what happens in the occidental world, where seaweeds are still under-explored in food terms, oriental cultures have used this resource since ancient times. In fact, in Asian countries such as Japan, one-fifth of daily meals have seaweed or seaweed compounds [6,14,15,16]. However, in other parts of the world, the inclusion of seaweeds has traditionally been focused in small coastal areas that have, or have had, problems with a regular supply of food. Now, seaweeds are being inserted into western diets, and they are gaining more interest due to the health-food industry and a transition into nutraceuticals [16,17].

Bioactive compounds taken from algae have fuelled nutraceutical interest in seaweeds. Polysaccharides (for instance, alginate, fucoidan, ulvan, agar, and carrageenan), proteins (for example, phycobiliproteins), carotenoids (beta-carotene and fucoxanthin), phenolic compounds (such as phlorotannins), vitamins (particularly vitamins A, B, C, D, E, and K), essential minerals (such as calcium, iron, iodine, magnesium, and potassium) and polyunsaturated fatty acids (namely ω-3 fatty acids) [14,16,18,19,20,21] constitute the interesting group of compounds. These compounds, isolated from seaweed, have been studied against diseases and pathologies that directly affect humans, such as hyperglycemia, diabetes, metabolic disorders, cancer, pathogenic diseases, aging, obesity, bone-related diseases, and neurodegenerative and cardiovascular diseases [22,23,24,25,26,27].

Due to the general incremental interest in seaweeds, there is a need to limit the risks of damaging wild seaweed stocks in order to prevent the overexploitation of this natural resource. Looking for efficient aquaculture systems is a way to mitigate environmental pressure caused by the growing demand [28]. To surpass environmental factors, it is important to understand the profile of seaweeds, for example, seaweeds that can be harvested in the wild due to their fast growth, such as *Ascophyllum nodosum*, or farmed, because they grow better in aquaculture systems, such as *Saccharina latissima* [29]. The seaweed aquaculture can provide new ways to standardize or control the seaweeds’ nutritional value because wild seaweed presents a widely variable composition provided by their ability to rapidly adapt to abiotic and biotic factors. Under aquaculture conditions, nutritional values can be monitored and more standardized when compared with wild seaweeds. This control is more effective in land-based aquaculture but there has been recent investigations into offshore farms to see if it is possible to extrapolate the growth and nutritional values of seaweeds. The variation of the nutritional values is mainly dependent on the species cultivated, seasons, geography/location, seawater quality (nutrients available), and the influence of other environmental stress factors during growth (such as temperature, pH, conductivity, salinity, UV radiation, light, and herbivory) [13,30,31,32,33,34,35,36,37,38].

The seaweed market is expected to grow 8.9% per year until 2050; it was estimated at over 11.7 billion US$ in 2016, and the previsions are that by 2024 the seaweed market will be worth 22.13 billion US$. Therefore, seaweed aquaculture has been demonstrated as the most sustainable way of seaweed production, considering that it already accounted for 30,139 thousand tons of seaweeds produced in 2016, representing nearly 96% of the global seaweed market [39]. Along with the growing demand for seaweed and seaweed-based food ingredients, an important call for more established guidelines and regulations to ensure sustainability has emerged. It includes a manual of best practices, certification/validation of the seaweed quality, and an environmentally neutral and sustainable value chain within the agri-food sector, to prevent an environmentally and health-harmful seaweed aquaculture and respective value chain [15,16,40]. Globally, there is an emergence of worldwide accepted standards that are being developed, such as the Seaweed Standard of the joint Marine Stewardship Council and Aquaculture Stewardship Council to guarantee the reduction in the negative impacts of the seaweed aquaculture and shift the seaweed aquaculture in the direction of an environmentally and socially responsible food source [39,41]. These standards and directives have evolved in a close relationship to support a more ecofriendly approach [42,43,44,45,46,47,48].

The growing presence of commercial seaweed-based products has led to the targeting of seaweed or seaweed extracts by the market/producers as a valorized product, mainly in nutraceutical and food supplement segments. Currently, the benefits of seaweeds to human health, some of them supported by scientific publications, are gaining more impact on the present society. However, as seaweeds have a great ability to perform bioaccumulation of heavy metals, contaminants [49,50], and iodine [51], the concerns about the consumption of seaweeds are increasing, particularly regarding human health safety. This apprehension is specially related to heavy metals or metalloids (mainly, arsenic), harmful compounds, and iodine concentrations variability in seaweed, which can be very dangerous for human consumption in several species [16,52,53]. This problematic is derived from the reduced/lack of legislation to require food or supplement companies to have labels with information about the essential minerals and potential heavy metals’ content in the seaweed-based (and other) products or a description/observation of the safe portion of the product that can be taken without incurring in an excessive and harmful intake [16,17]. The World Health Organization has been alerting for the arsenic risks in the aqueous system, establishing a recommended guideline value of 10 μg/L of arsenic concentration in the water [54]. Moreover, the European Food Safety Agency has been working hard in this field to fully understand the seaweed thematic and its risks, to make the European countries take measures towards secure seaweed consumption, but there is a long road to obtain the complete labelling of the products, with more information [55,56,57,58]. Indeed, the European Union has been imposing restrictive maximum concentrations of diverse contaminants in the foodstuff, for example, lead, cadmium, mercury and arsenic [59,60]. Still, further food contaminants are expected to be analyzed, so that the European Union can publish concentration limits.

Therefore, as will be discussed further in this review, there is a need to control extraction, production and/or processing methodologies and analyse the possibility of adapting the way of cooking the seaweed, as well as certifying and standardizing products with these ingredients, finally classifying them as safe. Precisely, quality criteria, such as the maximum levels of toxic minerals, have been defined for the sea vegetables [61]. Seaweeds are included in the “novel foods” regulations in Europe [62], and in the USA the use of brown seaweeds, named “kelp” (*Laminaria* sp., *Macrocystis* sp.), as human food is authorized by the Food and Drug Administration (FDA) [61].

Global food security is a flexible operational concept at the definition and policy usage, which has been evolving for decades [63]. Food security is achieved when all people, at all times, have physical, social and economic access to safe, sufficient and nutritious food that can meet the individual dietary needs and food preferences of a healthy and active life. The parameter that serves as a guide for this measure is the sub-nutrition calculus: when the food intake is not enough to fulfill the energy requirements, there is a lack of food, or there is the ingestion of low-nutrient foods. Food security has four correlated elements that are [64]:

- Availability of food supply with sustainable methods of farming,
- Physical and economic access to food,
- Utilization, that is, how the body uses the nutrients in food, how the food is prepared, and better food utilization is needed to prevent post-harvest losses,
- Stability of the food access, both economically and physically.

If there is access to quality and nutritious food, this will produce a positive impact in the world: for example, high improvement of the health and healthcare of the human population, but also economic growth, job creation, poverty reduction and an increase in trade opportunities, improving global security and stability [63,65].

Seaweeds can assume an important role in global food security, being a nutritious food when produced and consumed within the safety standards. Still, further advances are needed to sustain seaweed as a key element in food security, at the level of actively monitoring the potentially harmful compounds, developing and increasing seaweed aquaculture, and developing new processing methods to ensure the risky compounds are below the harmful level, thus protecting human consumption and being more available in the market [21,66,67]. On one hand, a controlled aquaculture system can be the key for safe seaweed production to cultivate more standardized seaweeds with a strict control of the seaweed quality. On the other hand, the other hypothesis is to do what is already happening with some compounds: a specific extraction and various assay tests to take data and develop regulation as to the hydrocolloids that seaweeds have [55,56,68,69].

The seaweed market is now a sector growing mostly under the blue economy and the flagship of the SDGs by countries to promote more companies and start-ups to take a bow for a blue and circular economy, encouraging the creation of more sustainable businesses and the change of mindset, to reduce the danger of environmental collapse [4,70]. This blue economy can target food security in developing countries, whose economy cannot hold a high price solution without an economic return. Seaweed aquaculture can be more stable, ecologically and environmentally neutral and economically viable than fishing [6]. Moreover, seaweed cultivation depends on low economic profile factors at the main basis, such as sunlight, seawater with inorganic nutrients and carbon dioxide [6].

There has been development and research of seaweed aquaculture aiming to understand seaweeds and their nutritional quality factors because the wild seaweed is not the best choice due to its high variability in composition [70,71].

This work discusses seaweeds from a nutritional point of view, more intrinsically the most common and most consumed seaweed species in the world, focusing on their nutritional profile and on food security. This review also highlights the seaweeds’ compounds that have already been legalized to be commercialized and applied in food industry, as food additives or food supplements.

## 2. Seaweed Nutritional Characterization and Daily Reference Intake

Seaweeds have been a mark of the Asian culture and cuisine for millennia. Indeed, China and Japan continue to be the countries with the highest consumption of algae in food, although they are also consumed, in a lesser extent, in other areas of the globe, particularly in coastal populations [14,72]. These sea vegetables are part of the diet of the people from the Pacific Islands and Asia, being served as snacks, side dishes, desserts and part of salads or even added as flavouring to noodles, soups, stews, garnishes and drinks [73,74]. Edible seaweed products are available in a variety of forms, either fresh or dry, powdered or flaked [75,76]. It is difficult to quantify the real amount of seaweed consumed, but the average estimate of seaweed intake in Japan is 4–7 g dry weight (DW) per capita per day [77,78,79,80].

There are over 600 edible seaweed species recognized worldwide, *Porphyra/Pyropia* sp., *Undaria pinnatifida* and *Saccharina*/*Laminaria* sp. being the top three applied in Asian meals, respectively known as food product names “nori”, “wakame” and “kombu” [81]. Although their compositions vary widely, depending on the species, location and growing/production conditions, and harvest season, they are all capable of providing important macro- and micronutrients indispensable for good nutrition. Macronutrients are the ones that need to be ingested daily in larger amounts, supplying energy, and they are proteins, lipids and carbohydrates. Micronutrients include vitamins, minerals and trace elements, which, despite being needed in very small quantities, are indispensable for the maintaining of vital functions [82].

Countries and continental organizations have made an effort to establish and regulate the patterns of the daily nutritional needs. For instance, in European Union countries, the Nutrient Reference Values (NRV) are stated for a healthy daily diet regarding the total of 8400 kJ/2000 kcal that should be ingested per day by an adult to maintain a healthy state (Table 1, Table 2, Table 3 and Table 4) [83]. In addition, the Standing Committee on the Scientific Evaluation of Dietary Reference Intakes of the Food and Nutrition Board of the National Academy of Sciences (NAAS) of the USA, with the participation of Health Canada, has established the daily nutritional needs for each age group, gender and state of pregnancy or lactating. In this case, the Dietary Reference Intake (DRI) was defined, taking into account the Recommended Dietary Allowance (RDA), Estimated Average Requirement (EAR), Adequate Intake (AI) and Acceptable Macronutrient Distribution Ranges (AMDR). RDA is the average sufficient daily dietary intake level to meet the nutrient requirements of nearly all (97–98%) healthy individuals of a group. It is derived from an EAR, which is an estimate of the intake at which the risk of inadequacy to an individual is 50%. However, if the scientific evidence is insufficient to establish an EAR, and consequently calculate a RDA, an AI is usually developed. AI is the daily quantity of dietary intake believed to cover the needs of all healthy individuals in a group, but lack of or uncertainty in the data prevents the specification of the percentage of individuals covered by this intake. Still, according to the NAAS, the AMDR is the range of macronutrients that should be uptake from the diet to ensure a reduced risk of chronic diseases and provide essential micronutrients, such as vitamins and minerals [82,84].

Seaweeds are considered a nutrient-rich dietary source of minerals, vitamins and dietary fiber (Table 5) [85,86,87,88]. Besides, they are a good source of protein, essential amino acids and compounds with antioxidant and anti-inflammatory properties, such as polyphenols [89]. Low lipid content, nonetheless enriched in ω-3 and ω-6 polyunsaturated fatty acids, makes seaweeds even more attractive, as they are a healthy, nutritive and low-caloric food [86,87].

Similarly, as they are a traditional food source, there are also historical records of the inclusion of seaweeds in folk medicine, mainly in Asia [90]. Thus, studies have successively demonstrated the importance of seaweed intake for human health, reporting beneficial effects on blood pressure [91], in decreasing the risk of ischemic heart disease [80], and even related with a decreased incidence of depression symptoms [92]. Besides that, there are plenty of worldwide in vitro and in vivo research studies verifying biological activities of seaweeds and their compounds, such as anti-inflammatory, neuroprotective, anti-cancer, anti-obesity, anti-viral, among others [23,53,93,94,95,96,97,98,99,100]. All this is gathering interest for seaweed cultivation and value-added seaweed-derived products for functional food and nutraceuticals in terms of improving human health status.

Given this, and adding the fact that we are facing a challenge in feeding a growing population on a planet where freshwater reserves are limited, arable land is scarce and intensive farming pollutes the soil with harmful chemicals, seaweeds are a sustainable alternative for feeding and meeting the nutrition aims of the world’s population.

### 2.1. Proteins

Proteins are macromolecules that perform diverse functions in living beings. They act as building blocks of bones, muscles, cartilage, skin, and blood, being crucial precursors of other molecules such as enzymes, antibodies and hormones [81,82]. Yet, unlike the other macronutrients (fat and carbohydrates), the body does not store protein and therefore has no reservoir to draw on when it needs a new supply. For instance, for an average adult, the daily ingestion of 50–56 g of protein is recommended (please see Table 1) [82,83]. Protein is crucial in everyone’s diet, but it is especially important for athletes, who need to repair and build muscle tissue broken down during exercise. Thus, the American College of Sports and Medicine endorses a daily consumption of 1.2 to 1.7 g protein per kg of body weight [102].

In westerns diets, animal-origin products and legumes are the typical protein sources; however, seaweeds could be an alternative to these products. In general, proteins are the third most abundant class of molecules in seaweed’s composition, just behind carbohydrates (with the dietary fibers) and minerals. Nevertheless, depending on the species, geographical location and season of growth, the protein content of seaweeds may vary from 3 to 47% [94,103,104,105]. Studies revealed that seaweed’s protein content variations are related with the nitrogen bioavailability in seawater, so usually, protein levels are higher during the winter and at the beginning of spring, and lower during summer and early autumn [106,107,108].

Typically, among algae, red seaweeds present the highest amount of proteins (14–47 g protein/100 g of dry weight), whereas brown have the lowest (7–16 g/100 g of dry weight), except for *Undaria pinnatifida* (“wakame”), which has an upper protein content (11–24 g/100 g of dry weight) [94,103,105,109]. Green algae generally have intermediate levels of protein (7–27 g/100 g of dry weight), reaching up to the lower values recorded in most red algae [104,110,111].

Moreover, seaweeds are complete protein sources, because they contain all nine essential amino acids (EAAs): histidine, isoleucine, leucine, lysine, methionine, phenylalanine, threonine, tryptophan, and valine [82,94,108], which are vital for protein synthesis, tissue repair and nutrient absorption. To guarantee the proper functioning of the organism, we have to uptake EAAs through our diet, since we are not capable of synthesizing them [82]. Regardless of total protein levels, all seaweeds account for up to 50% of the total amino acid content, where tryptophan, methionine and leucine are the principal limiting AAs in algae protein for most species [94,105,108,111,112]. Thus, seaweeds are a valuable source of protein, not only for total protein content, but mostly by their composition of amino acids [111]. The genus *Porphyra*/*Pyropia* has high protein levels (ranging from 26 to 48% in dry weight) [105,110,113], which are comparable to egg, soybean plant and even fishmeal, in terms of EAAs composition [103,108]. According to Wong and Cheung [111], the two red seaweeds *Hypnea japonica* and *H. charoides* and the green *Ulva lactuca* have, respectively, 424, 425 and 376 mg of total EAAs (except tryptophan, which was not determined in the study) per g of protein, thus presenting values over the 320 mg total EAAs per g of protein required by the Food and Agriculture Organization (FAO) [111].

In terms of non-essential amino acids (NEAAs), seaweeds share very similar patterns, in which aspartic and glutamic acid constitute a substantial fraction (20–32%) of total amino acids [86,105,111]. In fact, high concentrations of these two amino acids are responsible for the typical taste and “umami” flavour of the seaweeds [81,111].

Therefore, seaweeds are an excellent source of complete protein, since they have all the EAAs and also NEAAs. However, it is necessary to take into consideration their digestibility, as it can affect the number and type of amino acids effectively obtained by the human body [1,82].

For example, in vitro assays revealed digestibility of 78% of *Pyropia tenera*, 87% of *Undaria pinnatifida* and 95% of *Ulva australis* (formerly *Ulva pertusa*) proteins, wholly expressed as a percentage over casein’s (maximum digestion, 100%) [30,103].

However, the values of digestibility of algal proteins presented are high and could be compared to those reported for typically consumed land plants, including grains (69%–84%), legumes (72%–99%), fruits (72%–92%) and vegetables (68%–80%) [30].

Seaweeds’ high amount of phenolic compounds, more precisely tannins, and polysaccharide contents are the main factors that lead to algae proteins’ digestibility reduction, due to their ability to bind proteins and form insoluble complexes [114,115,116]. This could be overcome by heating the seaweed biomass (e.g., boiling), which causes partial denaturation and breakdown of proteins into smaller peptides, allowing easier access by the proteolytic enzymes [117,118].

### 2.2. Lipids

Lipids are macronutrients essential for our health when ingested in adequate amounts and with other nutrients. Their main function is to provide energy, being also indispensable in the formation of cell membranes and hormones [119]. They play an important role in transporting and absorbing fat-soluble vitamins (A, D, E and K) too. However, the excess of fat is related to a high prevalence of obesity, diabetes, dyslipidaemia and cardiovascular-associated co-morbidities [27,120].

Seaweeds are known as low-calorie food, mainly due to their low content in lipids (0.5%–4.5% of dry weight) [101,105,121]. Aside from being present in low quantity, it is important to distinguish them according to their chemical features. Seaweeds are composed of hydrocarbons (e.g., squalene), sterols (e.g., cholesterol) and mainly of all types of fatty acids (FA) whose abundances are variable within species, environmental conditions and life cycle phases [52,81,100]. The major factor that influences the FA composition is the temperature [122]. Thus, comparatively, tropical species contain fewer lipids than the cold water species [123].

Predominant FA found in the three groups of studied seaweeds were myristic acid (C14:0) and palmitic acid (C16:0), their respective monounsaturated variants, palmitoleic acid (C16:1) and oleic acid (C18:1), and linoleic acid (C18:3), the most abundant PUFA [100,123,124,125].

Indeed, palmitic acid is most of the times the major fatty acid component, ranging from 28.36 to 64.67% of total FA content, whereas palmitoleic acid varies from 1.0 to 19.51% and oleic acid from 4.0 to 17.3% [100,106,125].

A large lipid fraction (up to 74%) of marine algae is composed of ω-3 and ω-6 polyunsaturated fatty acids (PUFAs) [100,105,126]. These compounds are involved in many biological activities in the human body and are precursors of important compounds. Linoleic acid (LA, C18:2ω6) and α-linolenic acid (ALA, C18:3ω3) are the essential FA, which can be elongated to synthesize the remaining long-chain ω-6 and ω-3 PUFAs, and are also hormonal processors, including eicosanoids, in the human metabolism [123,127]. Commonly, green algae present interesting levels of ALA [100,123].

For instance, in seaweeds, LA may represent between 1.03 and 4.65% of total FA content of a species, ALA occurs from 0.1 to 11.7%, arachidonic acid (ARA, C20:4ω6) from 1.2 to 9.8%, and eicosapentaenoic acid (EPA, C20:5ω3) from 1.07 to 9.89%. Green algae *Ulva clathrata* (formerly known as *Enteromorpha clathrata*) was characterized by a high amount of LA (16.7% of total FA) whereas the red macroalgae *Acanthophora spicifera* contained large amounts of EPA (13%) and oleic acid (11%) [100,123].

Both ω-3 and ω-6 PUFAs are essential to humans and must be obtained from the diet in a balanced proportion. The ideal ratio of ω-6:ω-3 should range from 3:1 to 5:1 and it is associated with a reduction in the risk of cardiovascular and other chronic diseases, such as diabetes, and the improvement of the immune response and brain function [119,128,129]. On average, this ratio ranges from 0.1 to 8.2 in red species, from 0.2 to 2 in green species, and 0.2 to 2.4 in brown species [52,101,106,130,131,132,133,134]. Thus, consumption of seaweed products should be encouraged as they offer these health-promoting PUFAs.

### 2.3. Carbohydrates

Carbohydrates are highly present in seaweeds (in some species, this fraction represents over 50% of their dry weight), functioning as a photosynthetic reserve and as osmoregulators. They are a very heterogeneous group of macronutrients, including sugars, starches and fibers [81,135].

Simple carbohydrates (monosaccharides, also called sugars) are fast-absorbed nutrients, constituting the body’s main energy source. They provide the fuel for the central nervous system and energy for working muscles. They also prevent protein from being used as an energy source and enable fat metabolism.

These low molecular weight carbohydrates are predominant regarding the seaweed group and their production varies according to abiotic conditions during the seaweed growth. For example, a sugar present in green algae is sucrose [136], while red algae present floridoside, isofloridoside and digeneaside [137,138,139]. In addition, mannitol, which is an authorized food and drug ingredient, can represent 3–30% of brown seaweed’s dry weight [131,140,141,142,143]. However, when ingested, only 25% of mannitol is absorbed by the organism and thereafter completely excreted in the urine; the remaining 75% is fermented by the microbiota [135]. Despite the presence of these low molecular weight molecules, the most representative carbohydrates in seaweeds are the long-chain polysaccharides (composed by more than ten monosaccharides) [144].

There are two main types of polysaccharides in seaweeds—structural and storage. Therefore, they behave as fibers and fit in a distinct nutritional category: dietary fibers [77,145].

#### Fibers

Unlike sugars and starches, fibers are not absorbed in the small intestine, nor converted to glucose, thus they do not provide energy [145]. As these long-chain molecules are indigestible by the human body due to the lack of enzymes capable of disrupting the glycosidic bonds of the polysaccharides, they develop different roles in the organism [145].

However, the structural polysaccharides from seaweeds are nutritionally important, since they promote a sense of satiety, regulate the intestinal function, modulate the microflora of the intestine and have the ability to alter the absorption rates of other nutrients, as a result of a complete or partial fermentation in the large intestine [86,146,147,148,149].

Structural polysaccharides are analogous to terrestrial plants and are mainly celluloses, hemicelluloses, xylans and mannans, while storage polysaccharides, such as carrageenan, alginate, and agar, are more specific to seaweed species, and represent the most commercially exploited components in seaweeds [69,150]. These storage polysaccharides exhibit textural and stabilizing properties, being widely extracted by the hydrocolloid industry and used in food applications [121].

Major seaweed polysaccharides are group-specific. Thus, alginate (alginic acid) is the most abundant within brown seaweeds polysaccharides, reaching up to 70% DW [151,152], followed by other minor polysaccharides, such as laminarin and fucoidan [135,152,153].

Alginate content differs within brown algae species. For instance, *Sargassum baccularia*, *Sargassum aquifolium* (formerly known as *Sargassum binderi*), *Laminaria hyperborea*, *Turbinaria conoides* and *Sargassum siliquosum* show alginate yields of 26.7%, 38.7%, 40.8%, 41.4% and 49.9% of the seaweeds’ dry weight, respectively [154,155].

Particularly in Laminariales, such as *Saccharina latissima* (formerly known as *Laminaria saccharina*) and *L. digitata*, there is the polysaccharide laminarin, which can reach 35% (usually 10–30%) of the seaweed dry matter [143,156]. Additionally, when ingested, laminarin can be partially or completely fermented by the endogenous intestinal microflora [157].

On the other hand, fucoidan is the most representative in *Fucales*, in which it could range between 3.4 and 25.7% of dry biomass [158].

A distinctive polymer of green seaweed species is the polysaccharide ulvan, which may represent 8 to 29% of seaweed dry weight [159,160].

Red seaweeds have agar, carrageenan and porphyran as particular polysaccharides [24].

In short, seaweeds are rich in a variety of polysaccharides, since they are the main components of their cell walls. In addition, these organisms have greater fiber content than the terrestrial vegetables and fruits [86].

The soluble dietary fiber, mainly non-cellulosic polysaccharides and oligosaccharides, dissolves in water, forming a viscous gel that promotes a delay in gastric emptying, regulates blood glucose levels and lowers serum LDL cholesterol levels, due to its effect of increasing the viscosity of gut content and colonic fermentation, being important in pathologies like diabetes [86,149,161].

Whereas insoluble fiber comprises cellulose, hemicellulose and lignin, and plays an important role in bowel transit regulation and laxation due to its bulking capacity, it also supports the growth of intestinal microflora (probiotic species) due to its fermentation in the large intestine [94,147,148,149].

Furthermore, dietary fiber intake is associated with a lower risk of over-weight or obesity [162,163] and improves immune function through gut health and fiber–microbiota interactions, thus being partially or totally fermented by the endogenous intestinal microflora [157,164,165]. The FDA also approved two health claims regarding the dietary fiber physiological benefits for human health. The first claim states that, along with a decreased consumption of fats (<30% of calories), consumption of dietary fiber may reduce some types of cancer. The second is that diets low in saturated fat (<10% of calories) and cholesterol and high in fiber decreased the risk of coronary heart disease [161].

Consequently, the European Food Safety Authority (EFSA) recommends the ingestion of 25 g/day of dietary fiber [166].

Hence, although the amount varies within species and environmental conditions of growth, seaweeds are a great source of dietary fibers, as it is the case for *Undaria pinnatifida*, *Palmaria palmata* and *Ulva rigida*, that exhibit, respectively, 16–51%, 29–46% and 38–41% of dietary fiber by dry weight biomass [86]. It should be noted that oligo- and polysaccharides of seaweeds have several other interesting bioactivities, including anti-inflammatory, anti-bacterial, anti-viral, anti-diabetic, and immunomodulatory activities [167,168,169,170,171]. Maeda (2005) found that the ingestion of a daily agar supplement in human diets, as a jelly-like preparation containing 4.5 g of agar seasoned with condiments, results in weight loss, due to the maintenance of a reduced caloric intake, and causes an improvement in metabolic parameters, with no side-effects [171]. In addition, porphyran showed an anti-allergic effect, since the oral administration of 2% porphyran in drinking water, in mice with ear edema, caused a depletion in IgE and interferon-γ serum levels [169], demonstrating this particular bioactivity and oral food safety.

### 2.4. Vitamins

Vitamins, as micronutrients, are essential for the normal functioning of the body in very small amounts. They are classified according to their solubility. Fat-soluble vitamins are represented by vitamins A, D, E and K. These require the intake of adequate amounts of dietary fat, since lipids are indispensable to their absorption, transport and cellular uptake [172].

The other group comprises the water-soluble vitamins, which are vitamin C, vitamin B1 (thiamine), vitamin B2 (riboflavin), vitamin B6, vitamin B12, niacin, pantothenic acid, biotin and folate. These water-soluble vitamins are easily leached out of food products into the cooking water (e.g., boiling), but, then, they are not lost if the cooking fluids are consumed [172,173].

Vitamins are a diverse group of compounds in terms of molecular structure and physiological functions and are extensively disseminated in common food sources, including in seaweeds [143,172].

These micronutrients play a vital role in the maintenance of health as co-enzymes, precursors of hormones or antioxidants and thus have beneficial effects in the prevention of heart-related diseases, bone diseases and cancer [143,172,174,175,176]. Vitamins A and D have received particular attention in recent years as these vitamins have been shown to have a crucial effect on the immune system [84,177]. Deficiencies in these compounds, for instance in vitamin A, vitamin D, thiamine, niacin and vitamin C, can lead to several pathologies, respectively, xerophthalmia, rickets, beriberi, pellagra and scurvy [172].

Vitamins, except D and K, must be provided by the diet because they are not produced in adequate amounts by the human body. Vitamin D is formed in the skin through exposure to ultraviolet light, and can be supplied through diet, and vitamin K is produced by intestinal bacteria [172,178].

Seaweeds are rich in both water and fat-soluble vitamins, namely in A, B, C and E (Table 6) [77,121,179] and the production of these compounds depends on environmental factors, especially on their exposure to sunlight [121]. About 100 g of seaweed provides more than the daily requirement of vitamins A, B2 and B12 and two-thirds of the requirement of vitamin C. However, as the western gut is not historically adapted to seaweed ingestion, the introduction of this ingredient in the diet must be gradual, where the daily portion of 5 g has shown several health benefits, such as improving the immune function and reducing disease risks [16,180].

Vitamin A, which plays a significant role in reproduction, embryonic development, growth, immune function and in the maintenance of a normal vision, occurs in seaweeds such as *Fucus spiralis* (1.41 mg/100 g of dry weight), *Porphyra/Pyropia* spp. (1.27 mg/100 g of dry weight) and *Osmundea pinnatifida* (1.20 mg/100 g of dry weight) [84,181]. *Porphyra/Pyropia* spp. and *Ulva* spp. can have higher contents in vitamin C than an orange or a kiwi, in corresponding weights [143,181,182]. In addition, in 100 mg of dry seaweed, *Fucus spiralis* and *Gelidiella acerosa* have around 104 and 133 mg of vitamin D, respectively [121,183]. Vitamin B12, that has an RDA of 2.5 mg/day for adults, is abundant in species such as *Ulva* spp. and *Porphyra/Pyropia* spp. [82,121]. Being of major relevance, seaweeds are one of the few vegetable sources of vitamin B12, so they are an excellent option for vegetarians or vegans to get this essential nutrient and achieve its daily requirements [121].

### 2.5. Minerals and Trace Elements

Along with vitamins, minerals and trace elements constitute the micronutrients group. According to the World Health Organization (WHO), they are needed only in tiny amounts; however, they are essential to enable the body to produce enzymes, hormones and other substances crucial for proper growth and development. These molecules have numerous structural functions involving the skeleton and soft tissues, and regulatory functions such as neuromuscular transmission, blood clotting and oxygen transport [184]. Although required in small amounts, their unavailability leads to severe consequences.

Iron and iodine are responsible for two major public health harms caused by dietary micronutrients insufficiency intake. Iron (Fe) is an essential component of many proteins, including enzymes and haemoglobin present in red blood cells, being indispensable for binding and transport of oxygen throughout the body tissues [84]. A severe lack of dietary iron depletes iron stores in the body, and this can eventually lead to iron deficiency anaemia, that is considered the most common nutritional deficiency condition [185].

Iodine (I) is another essential micronutrient. It is a component of the thyroid hormones, thyroxine (T4) and triiodothyronine (T3), which are vital regulators of metabolic rate and physical and mental development. Iodine deficiency results in lethargy and swelling of the thyroid gland (hypothyroidism) in the neck which forms goiter. Infants born of severely iodine-deficient mothers may be mentally handicapped (cretinism) [186].

Also, Zinc (Zn) is necessary for development and growth [172]. Due to zinc’s ubiquitous involvement in metabolic processes, its deficiency has been associated with dysfunctions of epidermal, central nervous, immune, gastrointestinal, skeletal, and reproductive systems [19,84]. Calcium (Ca) and phosphorus (P) are crucial for the heart and smooth muscle contraction and the skeleton, while magnesium (Mg) is a very relevant cofactor of many enzymes, such as those involved in cellular respiration. Sodium (Na) is responsible for regulating body water content and electrolyte balance. High intake of Ca, Na and potassium (K) correlate with lower mean systolic pressure and lower risk of hypertension [123].

Due to their ability to absorb and accumulate these elements from their habitat, seaweeds are an extraordinary source of minerals and trace elements (8–40% of the dry weight) indispensable to the human diet, having a higher content of these compounds than the terrestrial or freshwater food products [77,88,143]. Although the seaweeds’ mineral profile varies according to species, the geographical place of harvest, wave exposure, seasonal, annual, environmental and physiological factors, type of processing and method of mineralization, they may be an important source of minerals (Table 7) to include in the human diet, since some of these trace elements are lacking or are present to a lesser extent in land vegetables [20,111,143,187,188]. Marine algae have high contents of these elements, namely I, Fe, Mg, Ca, P, Na and K [20,88,123]. A study with *Undaria pinnatifida* of two different origins, from Europe and Asia, demonstrates divergences in some mineral values. K and Mg were significantly different: the European algae was richer in K and poorer in Mg, when compared with the Asian “wakame” [188]. Biancarosa et al. [131] characterized several macroalgae from the Norwegian coast, *Ulva intestinalis* was the one that presented the highest content of Fe (5800 mg/Kg dry weight), *Fucus vesiculosus* had 30 g of Ca per Kg of dry algae, and brown seaweeds, such as *Saccharina latissima* and *Laminaria digitata*, were rich in I (respectively, 4600 and 10,000 mg/Kg of dry seaweed). Moreover, marine algae are, definitively, the best natural sources of bioavailable dietary I. According to the WHO and FAO, the dietary reference value for adults (>13 years) is 150 µg/day (i.e., 2.0 µg/kg/day) [19,84,94,186]. Furthermore, Narayan et al. [123] stated that seaweeds can contain 1000 times as much iodine as found in marine fish, such as cod. Seaweeds ingestion could be a way to treat several dysfunctions or attenuate pathological symptoms due to the lack or deficit consumption of minerals [77]. However, referring to iodine and other elements, bioaccumulation (e.g., inorganic arsenic) is the biggest issue that leads to a resistance to the use of algae in the diet [189,190].

Excess dietary iodine may lead to thyrotoxicosis and could be associated with hyperthyroidism [190,191]. So, ingestion of seaweeds, namely brown ones, that have higher contents of I, must have some precautions since the daily intake of more than 1100 µg (tolerable upper intake level for adults) may cause harmful effects [158,186]. Above the specific features and geographical and environmental factors, iodine has been reported to vary with age and condition of the seaweed, with iodine concentration dropping when the seaweed is no longer growing. On the other hand, there is a differential distribution of iodine in the seaweed, so usually, the stipe (stalk), where the meristematic tissue is and that is not commonly harvested and edible, at the base of the blade, has the highest iodine content [192]. Moreover, note that iodine is water-soluble, which means that preservation and cooking procedures affect seaweed iodine concentration of ingested food [52,192]. It was found that, when boiling *L. japonica*, it loses 99% of the iodine into the cooking water, resulting in a stock high in iodine [193]. In addition, boiled *Alaria esculenta* reduced the iodine content (from 670 mg/g to 165 mg/g of the dry biomass), as well as *Palmaria palmata* (97 mg/g to 66 mg/g) and *Ulva intestinalis* (92 mg/g to 79 mg/g) [194]. Thus, excess ingestion of high iodine-containing foods is highly inadvisable for susceptible subjects with thyroid dysfunctions as the hyperthyroidism [51]. However, by being aware and choosing the right seaweed and food processing/cooking methodology, anyone can eat seaweed without compromising their health [194]. In line with this, according to an individual’s iodine-related pathologies, and knowing that seaweed consumption may benefit an individual’s health, prevention of excessive iodine intake may include the disclosure of iodine content and the provision of cooking instructions on seaweeds’ product labeling to ensure consumer safety.

Another issue of seaweed ingestion is the chemical toxicity associated with the absorption and bioaccumulation of heavy metals from the environment, such as lead, mercury, cadmium and arsenic (inorganic arsenic) [195]. These values on seaweeds depend on the external contamination, which has led to inconsistency in research findings. For instance, on one hand, a Korean study was conducted to test 426 seaweed samples for arsenic, lead, mercury, and cadmium levels. Arsenic was highest at 17.4 mg/kg dry weight, and assuming 8.5 g per day seaweed consumption, the intake was falling in the WHO recommended weekly limit (0.2–6.7%). This study verified very low chances of health risks from the metals consumed via seaweed [196]. However, other works revealed that the consumption of inorganic arsenic increases the incidence of cancers and has also been linked to skin lesions, cardiovascular disease, neurological effects, and diabetes [84,197,198]. Arsenic occurs in both inorganic and organic forms, with the inorganic species (e.g., trivalent arsenite (III) and pentavalent arsenate (V)) having the greatest toxicological significance [84]. The Japanese seaweed *Sargassum fusiforme* (formerly known as *Hizikia fusiforme*), has 68.3 to 149 mg of total arsenic per Kg of dry weight, and from 41.6 to 117 mg of inorganic arsenic per Kg of dry algal weight, exceeding the maximum limit of inorganic arsenic admitted by France and the USA of 3 mg/kg [199].

The highest concentrations of arsenic in food are found in marine products, thus, this is not a new concern, neither is it exclusively related to the consumption of algae, since it also occurs in other foods, such as fish and seafood [84,200]. Although there is no UL established for arsenic, there is no reason for the consumption of organic arsenic in food or intake of inorganic arsenic through water supplies as there may be a risk of adverse effects [84].

Given these concerns, some quality criteria were established for the trading of sea vegetables. For instance, the maximum level for inorganic arsenic is 3 mg/kg (dry weight), for iodine is 2000 mg/kg (dry weight), for cadmium 0.5 mg/kg (dry weight), for mercury 0.1 mg/kg (dry weight) and for lead 5 mg/kg (dry weight) [59,201]. Additionally, dried seaweeds have to be submitted to microbiological tests, as *Salmonella* sp. (0 in 25 g of product) and *Staphylococcus aureus* (<100/g of product) [61,201,202].

Algae are one of the best approaches to address the nutritional deficiencies found in some of the current “western” daily meals, due to its wide range of constituents: minerals (iron and calcium), protein (with all essential amino acids), vitamins and fibers.

Above their demonstrated nutritional and nutraceutical value, commercialized macroalgae and macroalgae products must undergo thorough quality controls and regulations. European legislation includes seaweed in the “novel foods” category of its regulations and is also authorized the use of seaweeds as human food by the FDA [61].

## 3. Seaweed Compounds and Food Industry Application

There is a rise in consumer awareness worldwide regarding food security, which leads to a demand for natural and sustainable additives of food products [203].

In this context, seaweeds and their extracts are gaining interest on a global level, due to their widespread applications in several industries. Particularly in the food industry, seaweeds are a valuable resource due to their nutritional composition and bioactivities, thus promoting human health [204]. Despite this, investigation has been focused on specific seaweed species and a few compounds [52]. However, seaweeds and their derivatives are already applied in food products manufacture [71,205]. It is highlighted by the FAO [206] that, in 2012, 80% of worldwide seaweed feedstock production was towards the food industry, either to direct consumption or processed food products.

Algae are categorized into three main groups, which are: red (Rhodophyta), brown (Phaeophyceae) and green (Chlorophyta), and they comprise high ecological biodiversity [46] and a wide range of food resources. The most consumed seaweeds worldwide are brown (66.5%), red (33%) and green (5%) [125].

Seaweeds’ composition is enriched in bioactive compounds, hence with therapeutic and nutraceutical value [150], such as polysaccharides, phenolic compounds, pigments, protein and lipids [25]. For this reason, macroalgae are considered a health promoter, known as a functional food/ingredient [205], through their anti-inflammatory, antimicrobial, immunomodulatory, antidiabetic and antihypertensive bioactivities [207].

By contrast, between 1980 and 2003, 14 cases of death and 73 cases of sickness, possibly due to seaweed direct consumption, were reported (specifically seaweeds from genera *Caulerpa*, *Gracilaria* and *Acanthophora*) in a specific geographical area (Pacific Basin). However, the source of the toxins that led to the harmful effects in humans was considered dubious [190].

For this reason, seaweeds and their by-products must fulfill determined regulations to be available on the food market. For instance, in the European Union, according to EU Regulation 2015/2283 [208], before a novel food’s entrance into the market, it is necessary for it to be previously accepted by the Union. Currently, among approved novel food products, there are phlorotannins (extracted from *Ecklonia cava*) and fucoidan (extracted from *Fucus vesiculosus* and *Undaria pinnatifida*). Seaweeds also contribute to the food industry and security as food additives approved by the competent authorities. Therefore, under the Commission Recommendation (EU) 2018/464, the monitoring of the concentration of metals and iodine in seaweed and their derivative products is also advised [209]. Meanwhile, in the United States of America, seaweeds’ application in the food industry is regulated by FDA, within the Department of Health and Human Services (title 21, chapter 1B, part 182) [210].

However, the legislation differs between countries, namely in the mandatory monitoring and the concentration limits accepted for seaweed compounds [211].

### 3.1. Polysaccharides

Seaweed polysaccharides’ chemical structure is distinctive of taxonomic groups, differing in accordance with the species, the season and the respective extraction method [153,212,213,214]. For instance, alginate, fucoidan and laminarin are characteristic of the Phaeophyceae class; while carrageenan and agar occur in phylum Rhodophyta and ulvan is representative of phylum Chlorophyta. Among them, only alginate, agar, carrageenan and fucoidan are the polysaccharides more relevant to the food industry [20,215,216].

#### 3.1.1. Alginate

The polysaccharide alginate was first isolated in the 1880s. However, its large-scale production began in 1929 [52].

Several seaweed species of the class Phaeophyceae are big producers of alginate; however, the species selection as alginate source will depend on the country where each one is dominant [217]. For that reason, the commercially available alginate is mostly from the following brown seaweed species: *Laminaria hyperborea*, *L. digitata*, *Saccharina japonica* (formerly known as *Laminaria japonica*), *Ascophyllum nodosum*, *Macrocystis pyrifera*, *Ecklonia maxima*, *Lessonia nigrescens*, *Durvillaea antarctica* and *Sargassum* sp. [218].

There are several methodologies for alginate extraction; however, at an industrial scale, the aim is to achieve the highest yield at the lowest costs [219]. The extraction of this hydrocolloid involves several steps, namely: washing to remove impurities; a pretreatment with heated acid (hydrochloric acid or sulfuric acid at 60 °C for 24 h) to remove pigments, proteins and lipids [220,221]. Thereafter, there is a solid–liquid extraction, where the pellet is submitted to an alkaline treatment (sodium hydroxide at 60 °C for 2 h) followed by a centrifugation or filtration process. After sodium alginate precipitation, filtration is needed in order to finally dry or mill the sample for further application [69,222].

This phycocolloid has widespread biotechnological and industrial applications [223,224,225], assuming its importance in the food industry [69,204]. Alginate is catalogued as a non-organic compound and it is approved by the Food and Drug Administration (USA) and by the EFSA as a food ingredient [217]. In this context, alginate application and labeling in food products are regulated according to the Regulation of European Union Commission (1333/2008) as E400 (alginic acid), E401 (sodium alginate), E402 (potassium alginate), E403 (ammonium alginate), E404 (calcium alginate) and E405 (propylene glycol alginate) [226]. This regulation also defines the maximum authorized concentration of these food additives in the different food categories (Table 8). Among the 69 food categories in the European Union, alginic acid and its salts (E 400–E 404) are allowed at *quantum satis* in almost all of the food categories except for peeled, cut and shredded fruit and vegetables; jam, jellies, marmalade and sweetened chestnut puree; other similar fruit or vegetable spreads; processed cereal-based foods and baby foods for infants and young children; and dietary foods for infants for special medical purposes and special formulae for infants.

Thus, alginate has a high capability of retaining water and its gelling properties are not compromised with temperature. Therefore, it is a food ingredient used as thickener, gel, emulsifier, flavor enhancer and stabilizer [223].

Since alginate retains moisture and water, it is highly viscous and enables the thickness of food products, being an advantage in manufacturing food products manufacture such as jam, marmalade, sauces (e.g., mayonnaise or salad topping), syrup, ice-cream and cake icing [227,228].

From another perspective, alginate’s capability of chelating ion metals and forming viscous solutions [229] allows its application as a stabilizer in canned food, sauces, jam, ice-creams and in drinks, such as yoghurt, chocolate milk and fruit beverages [221,230].

Alginate syneresis is also already applied in the pastry and bakery industries due to its gelling and flavor-enhancing properties [231,232].

Moreover, alginate gel coatings can also contribute to increasing food products’ shelf lives (e.g., meat, fish and fruits), through the prevention of lipid oxidation [233,234], and reduce the probability of biological contamination [69,235].

The FDA (2014) [210] approved alginate as safe for human consumption after toxicological assays. Thus, ammonium, calcium, potassium and sodium alginates are considered generally recognized as safe, when given the evidence of good practices in their manufacture and used in certain concentrations, which are tabulated in the legislation according to the food product [217].

#### 3.1.2. Agar

Agar is a hydrocolloid characteristic from red seaweed that was first discovered around 400 years ago in Japan. The commercial and industrial interest on this compound, however, started in the last 160 years, leading to natural stocks depletion [236]. It was large-scale seaweed aquaculture that allowed the growth of the agar industry around 50 years ago [69].

The main seaweed species explored commercially for agar extraction are from the genus *Gelidium* (harvested) and *Gracilaria* (harvested or cultivated). However, the quality of the final product differs between those genera. For instance, agar extracted derived from *Gelidium* is considered more suitable for pharmaceutical applications due to its characteristics and high quality [219]. Still, agar extracted from genus *Gracilaria* is commonly used for the food industry. Thus, further research demonstrated that an alkaline pretreatment with sodium hydroxide (2–5%) at 85–90 °C for 1 h could enhance agar’s extraction yield and quality by the increase in its rheological properties [69].

Generally, the industrial extraction method applied to agar extraction from seaweeds begins with a pretreatment process of washing, followed by heat treatment of the algal biomass in an aqueous solution (between 2–4 h at 105–110 °C for *Gelidium* and 95–100 °C for *Gracilaria*), for further immediate filtration, while the extract is hot. At this point, agar is dissolved in the filtrate and when it cools down, it forms a gel structure. This gel could present a yellow or brownish color, due to the presence of soluble carbohydrates or proteins and salts that could be washed with distilled water by the freeze/thaw technique, to obtain a concentrated hydrocolloid with a clear color. Finally, the polymer could be dried in an oven with air circulation and then milled for further application [69,222,237]. Another alternative extraction technique that is appropriate at an industrial scale resembles the syneresis method, which consists of hydraulic pressure utilization to accelerate water removal from the hydrocolloid, now concentrated [238]. The main advantages of this methodology are the reduction in energy and water costs that were spent to concentrate and dry agar.

Among all the biotechnological and industrial applications, agar assumes its importance in the food sector. It was approved as safe for human consumption by the food authorities of the United States of America and the European Union. Despite agar (E406) integration in the list of approved food additives, its application in food products is regulated and limited by a threshold. After being evaluated by the Scientific Committee for Food (SFC) and by the Joint FAO/WHO Expert Committee on Food Additives, agar application is authorized in 70 food products, according to the Annex II of the European Union Commission Regulation nº 1333/2008, being regulated the maximum concentration of application according to the food categories (Table 9) [226]. It is estimated that 90% of agar production is forwarded to the food industry [69]. Agar is allowed in *quantum satis* in almost all food categories except for jam, jellies and marmalade and other fruit and vegetable spreads.

Agar is characterized for dissolving in boiling water, thus gelling at temperatures around 32° to 43 °C and only melting if heated up to temperatures higher than 85 °C [236]. The ability that agar has of forming gel within high temperatures, makes it an exceptional food stabilizer and thickener of the confection of desserts such as jellies, pies, donuts, fruit candies and cake icings [236,239]. Additionally, agar is relevant for the beverage industry, as a clarifying agent of beer and wine, but also to dairy products such as cream cheese and yoghurt as an emulsifier ingredient [240].

Several studies have been done according to the guidelines for testing of chemicals of the Organization for Economic Co-operation and Development (OECD), in order to provide evidence of the security of agar consumption [241].

A study validated by EFSA appoints that under the agar concentrations tested in mice and rats (up to 4500 or 2500 mg/kg by body weight per day, respectively) it did not present carcinogenic effects [242]. However, some cases were reported of harmful effects after agar consumption by humans. For instance, agar gastric bezoars were reported in women with ages above 64 years old with a clinical history of obesity and diabetes, after consuming deserts based on agar or drinking agar solutions [243,244]. However, these isolated cases were not considered relevant for the evaluation of food safety of agar as a food additive, due to the medical history of the patients [55].

#### 3.1.3. Carrageenan

The first utilization of carrageenan was for food application, in a North Atlantic island [245,246]. However, it was in the United States that the industrial and commercial application of carrageenan started in 1940 [245].

*Chondrus crispus* (Rhodophyta) was the first seaweed species used for carrageenan production; however, since 1970, aquaculture allowed the commercialization of different carrageenophytes in other parts of the world [69]. For instance, *Kappaphycus alvarezii* and *Eucheuma denticulatum* are economically important cultivated seaweeds for carrageenan exploitation, while cultivation methods of *Chondrus crispus* and other red seaweed species from the genera *Gigartina*, *Iridaea* or *Furcellaria* need to evolve in order to be feasible at an industrial level [246].

In hydrocolloid extraction industry, seaweeds’ pretreatment (washing and/or drying) is a general common step, followed by the reduction in the particle size and a depigmentation step, in order to obtain a clear color on the final product [246,247]. According to the Regulation of European Union Commission (231/2012) from the European Union Commission and approved by the Joint FAO/WHO Expert Committee on Food Additives, carrageenan extraction should be performed with water or an aqueous alkaline solution diluted with water. Regarding the legislation, the organic precipitant used should be methanol, ethanol or propanol. Subsequently, the target hydrocolloid may be recovered through alcoholic precipitation, by drum drying or by precipitation in aqueous potassium chloride and further freezing. However, only methanol, ethanol and isopropanol can be used for carrageenan precipitation and purification [248]. Furthermore, as a food security measurement, normally this carbohydrate is sterilized before application [246].

In the second method, the carrageenan is never actually extracted from the seaweed. Rather, the principle is to wash everything out of the seaweed that will dissolve in alkali and water, leaving the carrageenan and other insoluble matter behind. This insoluble residue, consisting largely of carrageenan and cellulose, is then dried and sold as semi-refined carrageenan (SRC). Because the carrageenan does not need to be recovered from the solution, the process is much shorter and cheaper.

The selection of the alkaline solution could be a competitive advantage in the food industry. For this reason, several patents were developed to optimize carrageenan quality [247].

Further research showed that, among several carrageenophytes, different conformations of carrageenan could be found in seaweed extracts [249] with different food applications. For instance, *iota*-carrageenan (ι) is characterized for producing gels in the presence of calcium salts, while *kappa*-carrageenan (κ) forms a rigid gel in the presence of potassium salts and *lambda*-carrageenan (λ) only produces a high viscous solution [69].

In the European Union, food additives are regulated under the Regulations nº 1333/2008 and 231/2012 [226,248]. Hence, as part of the food additives list, carrageenan and processed seaweed *Eucheuma* need, respectively, to be labeled as E407 and E470a in commercialized food products [55].

Commonly, carrageenan (E407 and E470a) is applied in food products in low doses and it is authorized by the Joint FAO/WHO Expert Committee on Food Additives and it is allowed to be used at *quantum satis* in almost all food categories, except in infant formula or for special medical purposes (1000 mg/L) [250]. Moreover, the US FDA maintains that the level at which carrageenan is used in foods to achieve functionality is safe and that no upper limit needs to be established [210].

Due to carrageenan’s chemical structure diversity, this biomolecule has several areas of application within the food industry. For instance, in the dairy industry, κ-carrageenan is applied due to its emulsifier properties, improving the quality of cheese and milk, along with ι-carrageenan and λ-carrageenan that are usually applied in condensed milk [56]. Besides the application of ι-carrageenan in beer and wines as a clarifier agent, dairy beverages and creams also benefit from kappa and lambda carrageenan addition to chocolate milk or milkshakes, as a stabilizer agent. In line with the beverage industry, this polymer is also authorized in nectar and flavored juices or spirituous drinks [251].

In the bakery and pastry industries, this hydrocolloid supplement guarantees moisture control, enhancing the texture of these products. Its thickening and gelling properties are widely applied to the confection of sweets and desserts like puddings, jellies and ice-creams [66].

The meat industry also relies on κ and λ-carrageenans in order to improve the texture of meat products, preventing water loss while cooking or to replace fat addition in sausages [69].

However, there is a gap that is necessary to tackle with further research, which is to determine the stability of carrageenan (E407 and E407a) in food products, namely the byproducts resulting from acidic degradation of these food additives. Despite this fact, the EFSA determined that there were no reported cases of allergic reaction in humans due to food products with carrageenan as a food additive [56].

#### 3.1.4. Fucoidan

Fucoidan was first discovered in 1913 [252] and, since then, several studies have been reported highlighting its bioactive properties [110,253,254,255].

Brown seaweeds are the main marine producers of this polymer including the following seaweed species: *Cladosiphon* sp.; *Macrocystis pyrifera*; *Ecklonia radiata*; *Seirococcus axillaris*; *Saccharina latissima*; *Fucus vesiculosus*; *F. serratus*; *Fucus evanescens*; *F. distichus*; *F. spiralis*; *Laminaria digitata*; *Dictyosiphon foeniculaceus*; *Dictyopteris delicatula*; *Undaria pinnatifida*; *S. japonica* (formerly known as *Laminaria japonica*); *Ascophyllum nodosum*; *Durvillaea potatorum*; *Sargassum* sp. and *Padina boryana* (formerly known as *Padina commersonii*).

However, the EFSA (European Union) and FDA (United States of America) only authorized fucoidan extracts with a determined chemical characterization and derived from the seaweeds *F. vesiculosus* and *U. pinnatifida*.

According to the regulations, the final product must follow the required specifications (e.g., color, pH, odor, moisture and minerals, metals and other polysaccharides concentration range); however, in the European guidelines, the extraction procedure is quite general. This extraction should not involve organic solvents, but instead an acid diluted with water and further filtration, concentration and drying process [256]. In Europe, fucoidan is ruled by the novel food regulation 2017/2470 [256] and could be applied as a food supplement, according to the Directive 2002/46/EC [257].

To the FDA regulation [258,259], fucoidan is a non-toxic bioactive compound and a food functional ingredient, which means that its consumption is beneficial to human health; however, its consumption is limited to a known threshold [260]. Thus, fucoidan assumes its importance in food products such as bakery, soups, snacks, dairy products and as a flavor enhancer that can be used up to 30 mg/serving. However, the preservation of fucoidan molecules’ structural integrity essentially depends on the extraction methodology which has a significant role in order to obtain specific biological activities and for elucidating structure–function relations [261].

Several studies have been conducted to prove the safety of fucoidan extracted from *F*. *vesiculosus* [262,263,264] and *U. pinnatifida* [265,266,267,268] for human consumption. In this context, multiple clinical trials, as well as in vitro studies, further corroborate the absence of negative effects of this compound [262,263,264,265,266,267,268].

### 3.2. Phenolic Compounds

There are several secondary metabolites, in which phenolic compounds play a key role in seaweed protection pathways against biotic (e.g., herbivory) and abiotic stresses [269,270,271]. Thus, macroalgae extracts rich in phenolic compounds present biological activities that are beneficial and relevant to the food industry [203]. Although there are different phenolic substance categories, phlorotannins are relevant to the growth of seaweed belonging to Fucales order as part of its cell wall [272].

It is reported in the literature that seaweeds’ extracts rich in phenolic substances can increase food products’ shelf life, through its antioxidant and antimicrobial bioactivities [203,273].

Among seaweeds’ polyphenols, the phlorotannin dieckol assumes its significance in the food industry, being considered a novel food by the EFSA.

#### Dieckol

Dieckol (a hexamer) is a phlorotannin present in the brown seaweeds *Ecklonia* species. Historically, *E*. *cava* is a seaweed introduced in Japanese and Chinese nutritional culture [69]. Thus, recently this phlorotannin was considered a novel food by the European Union, according to the Commission Regulation (EU) 2018/455 [274].

In accordance with the European novel food regulations, these must follow good practices of industrial production and a certification process through ISO 22000:2005. Generally, the industrial extraction method implies a drying and milling process, followed by an ethanolic extraction and, finally, the purification, filtration and concentration of the bioactive compound [275].

Currently, this compound is already incorporated in the food industry as a food supplement. For instance, SeaPolynol™, a seaweed phlorotannin-based product, where dieckol is the main component, is a widespread product available in American, European and Asian markets [276]. Regarding food security, the responsible European authorities considered *E*. *cava* phlorotannins safe for human consumption, establishing a threshold of daily intake according to the age of the consumer [275,277]

### 3.3. Pigments

Among chlorophyll, and other accessory pigments (such as carotenoids) and phycobilins, there are light-harvesting compounds characteristic of certain seaweed groups. Seaweeds are categorized taxonomically according to the main accessory pigments that they possess [25]. For instance, the main accessory pigments in green macroalgae (Chlorophyta) are chlorophylls, while phycocyanin, allophycocyanin, carotenes (α and β) and phycoerythrin are the most abundant in red seaweeds (Rhodophyta), whereas in brown macroalgae (Phaeophyceae), there are mainly β-carotene, fucoxanthin, zeaxanthin and violaxanthin [278]. There is a wide diversity of edible pigments that could be extracted from seaweeds. For instance, some of the mentioned compounds are high-value natural pigments, which are already applied in the food industry as colorants.

The importance of these natural colorants settles on consumer awareness about food additives’ safety [279].

Several chromatographic methods can be applied to extract important pigments from seaweeds, such as centrifugal partition chromatography to extract fucoxanthin from the brown macroalgae *Eisenia bicyclis* [280]. Moreover, counter-current chromatography can be employed to extract the same previously mentioned compound from the seaweeds *Saccharina japonica*, *Undaria pinnatifida* and *Sargassum fusiforme* [281]. Ultrasound-assisted extraction and ultra-filtration was, however, a successful approach to extract chlorophylls and carotenoids from *U*. *pinnatifida* [282]. Phycoerythrin is also an important pigment that is extracted from red seaweeds. For instance, *Kappaphycus alvarezii* is usually extracted by using a buffer phosphate coupled with a freeze–thawing process. However, a study reported that using ultrasonication was also an effective approach for this pigment extraction [283].

Thus, the consumption of natural pigments promotes human health, and, among seaweed pigment categories, there are several potential candidates as functional ingredients [284]. In this context, fucoxanthin has been highlighted in the food market [25].

#### Fucoxanthin

Fucoxanthin is a xanthophyll characteristic of brown seaweeds that was first isolated in 1914. Initially, this bioactive phytochemical was detected in macroalgae belonging to the genera *Dictyota*, *Fucus*, and *Laminaria* [285], but this pigment is present in several seaweeds such as *Ascophyllum*, *Himanthalia*, *Sargassum* or *Saccharina*. However, *Undaria pinnatifida* is the most used species at an industrial scale, due to its high content of fucoxanthin [286,287,288].

This pigment is present inside of cell chloroplasts, so the extraction method has to consider this aspect. Industrially, the solid–liquid extraction of fucoxanthin is performed with solvents such as n-hexane, methanol, DMSO, ethanol, petroleum ether, diethyl ether, dimethyl ether, acetone, or ethyl acetate and dried to a powder [289,290].

More recently, fucoxanthin raised the interest of food market due to its antioxidant properties and beneficial advantages to human health. Currently, fucoxanthin derived from the diatom *Phaeodactylum tricornutum* is authorized by the FDA (in the United States) and by Food for Specified Health Uses (in Japan), being available in the market as a food supplement [290]. Until now, the mentioned authorities did not evaluate the clinical evidence to prove the beneficial fucoxanthin properties. However, there is no record of harmful effects in human health through the consumption of food supplements based on fucoxanthin [291].

## 4. Conclusions

This review highlights that seaweeds can be a vital key to global food security. Despite the need of answers to the increasing alarm over health and to obtain a secure and stable food supply, it is imperative to develop new policies that will enhance the quality and safety of seaweeds and their sub-products to human consumption. Furthermore, it is essential to promote and improve the recommended human daily nutrition dose and increase the overall health status.

To successfully surpass these objectives with the mindset of global food security, it is necessary to enhance cooperation and coordination among the countries and seaweed producers/associations to strengthen the functions of national and international policies as a whole. This is due to the potential of seaweeds as high nutritional food that are an alternative to agricultural products, with a great advantage that they can be implemented in coastal and sea zones as they have the conditions to use their sea quota to start or expand their production of macroalgae.

Algae are one of the best approaches to address the nutritional deficiencies of the current food, due to their wide range of constituents: minerals (iron and calcium), protein (with all essential amino acids), vitamins and fibers.

However, there is a need for supervision of the negative impact of the seaweeds in the human diet, such as the excessive intake of iodine and arsenic, so there is a need to control the seaweed nutritional quality and the negative factors of seaweeds that is also required in regular agriculture.

The commercial sub-products of seaweeds, mainly the polysaccharides, have been under inspection and have restricted orders and directives to be considered food-safe ingredients. These results are a guarantee of food security; however, the evolution of science obliges the world to evolve, and so, what is secure today, may not be tomorrow, a derivative of understanding how the human body works and its requirements, and how food is processed in the gastric system.

## Figures and Tables

**Table 1:** Daily reference intakes of the macronutrients established by the European Parliament and the Council of UE: Nutrient Reference Values (NRV) for an adult. Dietary Reference Intakes (DRI): Recommended Dietary Allowances (RDA) and Acceptable Macronutrient Distribution Ranges (AMDR) of macronutrients for a reference adult, according to the Food and Nutrition Board, Institute of Medicine, National Academies, USA. Adapted from [82,83]. ND—Not determined.

	Protein	Carbohydrate	Sugars	Total Fiber	Total Lipid	Saturated Fat	ω-6 Unsaturated Fat	ω-3 Unsaturated Fat
NRV (g) (EU)	50	260	90	ND	70	20	ND	ND
RDA (g) (USA)	56	130	ND	ND	ND	ND	ND	ND
AMDR (% of energy) (USA)	10–35	45–65	ND	38–30	20–35	ND	5–10	0.6–0.1

**Table 2:** Daily reference intakes of vitamins established by the European Parliament and the Council of UE: Nutrient Reference Values (NRV) for an adult. Dietary Reference Intakes (DRI): Recommended Dietary Allowances (RDA) and Adequate Intakes (AI) of vitamins for a reference adult man, according to the Food and Nutrition Board, Institute of Medicine, National Academies. Adapted from [82,83]. ND—Not determined. The * in front of values indicate that they are AI values.

	Vitamin A (µg)	Vitamin C (mg)	Vitamin D (µg)	Vitamin E (mg)	Vitamin K (µg)	Thiamin (B1) (mg)	Riboflavin (B2) (mg)	Niacin (mg)	Vitamin B6 (mg)	Folate (µg)	Vitamin B12 (µg)	Pantothenic Acid (mg)	Biotin (µg)	Choline (mg)
NRV (EU)	800	80	5	12	75	1.1	1.4	16	1.4	200	2.5	6	50	ND
RDA/AI * (USA)	900	90	15	15	120 *	1.2	1.3	16	1.3–1.7	400	2.4	5 *	30	550 *

**Table 3:** Daily reference intakes of minerals and trace elements established by the European Parliament and the Council of UE: Nutrient Reference Values (NRV) for an adult. Dietary Reference Intakes (DRI): Recommended Dietary Allowances (RDA) and Adequate Intakes (AI) of minerals and trace elements for a reference adult, according to the Food and Nutrition Board, Institute of Medicine, National Academies. Adapted from [82,83]. The * in front of values indicate that they are AI values.

	Calcium	Chromium	Copper	Fluoride	Iodine	Iron	Magnesium	Manganese	Molybdenum	Phosphorus	Selenium	Zinc	Potassium	**Sodium**	**Chloride**
	(mg)	(µg)	(µg)	(mg)	(µg)	(mg)	(mg)	(mg)	(µg)	(mg)	(µg)	(mg)	(mg)	**(mg)**	**(mg)**
NRV (EU)	800	40	1000	3.5	150	14	375	2	50	700	55	10	2000	600	2.4
RDA/ AI * (USA)	1000–1200 *	35–30 *	900	4 *	150	8	400–420	2.3 *	45	700	55	11	3400 *	1500 *	2.3–2.0 *

**Table 4:** Tolerable Upper Intake Levels (UL) for a reference adult. Adapted from [82].

Performed Vitamin A	Boron	Copper	Iodine	Iron
(µg/d)	(mg/d)	(µg/d)	(µg/d)	(mg/d)
3000	20	10,000	1100	45

**Table 5:** Nutrient composition of selected edible seaweed (% dry weight), adapted from [86,101]. ND—not determined.

Specie	Protein	Carbohydrate	Dietary Fiber	Lipid
Ochrophyta, Phaeophyceae (brown seaweed)				
*Fucus vesiculosus*	3–14	46.8	45–59	1.9
*Saccharina japonica*	7–8	51.9	10–41	1.0–1.9
*Sargassum fusiforme*	11.6	30.6	17–69	1.4
*Undaria pinnatifida*	12–23	45–51	16–51	1.1–4.5
Rhodophyta (red seaweed)				
*Chondrus crispus*	11–21	55–68	10–34	1.0–1.3
*Gracilaria chilensis*	13.7	66.1	ND	1.3
*Palmaria palmata*	8–35	46–56	29–46	0.7–3
*Neopyropia tenera*	28–47	44.3	12–35	0.7–1.3
Chlorophyta (green seaweed)				
*Caulerpa lentillifera*	10–13	38–59	33	0.9–1.1
*Codium fragile*	8–11	39–67	5.1	0.5–2.3
*Ulva compressa*	21–31	48.2	29–45	0.3–4.2
*Ulva lactuca*	10–15	36–43	29–55	0.6–1.6

**Table 6:** Vitamin composition of seaweed (mg/100 g dw). Adapted from MacArtain et al. and Syad et al. [121,183].

Seaweed	Vitamin A	Vitamin C	Vitamin D	Vitamin E	Vitamin K	Vitamin B1	Vitamin B2	Vitamin B6	Folate (g/kg)	Vitamin B12 (µg/kg)
Ochrophyta, Phaeophyceae (brown seaweed)										
*Ascophyllum nodosum*	-	81.8		-	-	27	7.3	0.125	0.456	16.4
*Fucus spiralis*	1.41	-	104	-	Trace	-	-	-	-	-
*Laminaria digitata*	-	355.3	-	-	-	1.38	1.38	64.1	0	61.9
*Sargassum wightii*	0.40	506.9	-	135	-	Trace	Trace	-	-	-
*Undaria pinnatifida*	-	1847	-	-	-	50.4	117	32.4	0.066	43.1
Rhodophyta (red seaweed)										
*Gelidiella acerosa*	0.34	507.18	133	-	-	Trace	Trace	-	-	-
*Osmundea pinnatifida*	1.2	-	-	-	2.56	-	-	-	-	-
*Palmaria palmata*	-	690	-	-	-	3	10	0.25	0.00263	230
*Porphyra umbilicalis*	-	1610.6	-	-	-	9.63	34.3	14.9	0.125	96.1
Chlorophyta (green seaweed)										
*Ulva rigida*	0.29	94.2	-	-	-	4.7	1.99	< 0.1	1.08	60
*Ulva* spp.	-	1250	-	-	-	7.5	3.75	0	0.0015	787.5

**Table 7:** Mineral composition of some edible seaweeds (mg/100 g dry weight), adapted from [86,131,188].

Species	Calcium	Copper	Iodine	Iron	Magnesium	Manganese	Phosphorus	Zinc	Potassium	Sodium
Ochrophyta, Phaeophyceae (brown seaweed)										
*Fucus vesiculosus*	725–3000	<0.5	14.50–26	29–11	670–994	3.7–5.50	100–315	3.71	2500–4322	1800–5469
*Saccharina japonica*	225–910	0.25–0.40	130–690	1.19–43	550–757	0.13–0.65	150–300	0.89–1.63	4350–5951	2532–3260
*Sargassum fusiforme*	1860	ND	43.6	88.6	687	ND	ND	1.35	ND	ND
*Undaria pinnatifida*	331–1380	0.19–2.0	22–30	1.54–30	277–680	0.27–0.56	235–450	0.94–4.03	864–6810	1600–7000
Rhodophyta (red seaweed)										
*Chondrus crispus*	420–1300	<0.5–0.76	24.5	4–20	600–900	1.32–2.2	135–240	7.14	1350–3184	1200–4270
*Palmaria palmata*	250–1200	<0.5	10–100	7.30–50	120–610	0.41–1.14	210–235	2.86	2800–9000	320–2500
*Neopyropia tenera*	390	0.63	1.7	10–11	565	3	ND	2–3	3500	3627
Chlorophyta (green seaweed)										
*Caulerpa lentilifera*	780–1874	0.11–2.20	ND	9.30–21.40	630–1650	7.90	700–1142	2.6–3.5	700–1142	8917
*Ulva lactuca*	840–1600	0.71	0.43	66–180	2700	2.6	140–220	ND	2800	700

**Table 8:** Food and Drug Administration (FDA) and European Food Safety Authority (EFSA) specific limitations of E400-E405 food additives in food products. *n.d.: nothing to declare.

Food Categories	Ammonium Alginate (%)		Alginic Acid (%)		Potassium Alginate (%)		Sodium Alginate (%)		Propylene Glycol Alginate (%)		Calcium Alginate (%)	
	FDA	EFSA	FDA	EFSA	FDA	EFSA	FDA	EFSA	FDA	EFSA	FDA	EFSA
Confections, frostings	0.4	n.d	0.3	n.d	n.d	n.d	0.3	n.d	0.5	n.d	0.4	n.d
Fats and oils	0.5	n.d	n.d	n.d	n.d	n.d	n.d	n.d	1.1	n.d	0.5	n.d
Gelatines, puddings	0.5	n.d	4	n.d	n.d	n.d	4	n.d	0.6	n.d	0.25	n.d
Gravies and sauces	0.4	n.d	n.d	n.d	n.d	n.d	n.d	n.d	0.5	n.d	0.4	n.d
Jams and jellies	0.4	1	n.d	1	1	n.d	n.d	1	0.4	-	0.5	1
Sweet sauces	0.5	n.d	n.d	n.d	n.d	n.d	n.d	n.d	n.d	n.d	0.5	n.d
All other food categories	0.1	n.d	1	n.d	n.d	n.d	1	n.d	0.3	n.d	0.3	n.d
Pimento ribbon for stuffed olives	n.d	n.d	6	n.d	n.d	n.d	6	n.d	n.d	n.d	n.d	n.d
Condiments and relishes	n.d	n.d	1	n.d	n.d	n.d	1	n.d	0.6	n.d	n.d	n.d
Hard candy	n.d	n.d	10	n.d	n.d	n.d	10	n.d	n.d	n.d	n.d	n.d
Processed fruits and fruit juices	n.d	n.d	2	n.d	n.d	n.d	2	n.d	n.d	n.d	n.d	n.d
Ice-creams	n.d	n.d	n.d	n.d	n.d	n.d	n.d	n.d	0.5	n.d	n.d	n.d
Cheese	n.d	n.d	n.d	n.d	n.d	n.d	n.d	n.d	0.9	n.d	n.d	n.d
Adjuvant in seasonings and flavours	n.d	n.d	n.d	n.d	n.d	n.d	n.d	n.d	1.7	n.d	n.d	n.d
Alcoholic beverages	n.d	n.d	n.d	n.d	n.d	n.d	n.d	n.d	n.d	n.d	0.4	n.d
Baked goods	n.d	n.d	n.d	n.d	n.d	n.d	n.d	n.d	n.d	n.d	0.002	n.d
Egg products	n.d	n.d	n.d	n.d	n.d	n.d	n.d	n.d	n.d	n.d	0.6	n.d
Peeled, cut and shredded fruit and vegetables	n.d	n.d	n.d	n.d	n.d	n.d	n.d	0.24	n.d	n.d	n.d	n.d
Cereals and foods for babies	n.d	n.d	n.d	0.05	n.d	0.05	n.d	0.05	n.d		n.d n.d	0.05

**Table 9:** FDA and EFSA specific limitations of agar (E406) as a food additive in food products. *n.d.: nothing to declare.

Food Categories	Agar (%)	
	FDA	EFSA
Baking goods	0.8	n.d
Confections and frostings	2	n.d
Soft candy	1.2	n.d
All other food categories	0.25	n.d
Other similar fruit or vegetable spreads	n.d	1
Jam, jellies and marmalades and sweetened chestnut puree	n.d	1
